# Orleans Parish Medical Society

**Published:** 1894-01

**Authors:** 


### *SOCIETY REPORTS.*

### ORLEANS PARISH MEDICAL SOCIETY.

New Orleans, October 14, 1893.

The regular monthly meeting of the Orleans Parish Medical Association was called to order by Vice-President Lowe.

Dr. Martin read a paper on

### LONG CONTINUED FEVERS IN LOUISIANA.

My desire this evening is to prompt a discussion on a subject which, though not entirely new to the Society, is, I believe, most deserving of consideration. I have had to cope recently with a type of fever foreign to any represented in our text-books. From older practitioners I learn that these slow fevers of Louisiana, which resist all forms of treatment, and which are sometimes more aggravated than benefited by quinine, are of recent origin in this State. With the many forms of malarial fever we are thoroughly conversant, though it is my belief that the prefix "malaria" is too often used to supply a want in the absence of a diagnosis. In my own brief experience I have known a case of abscess of the liver to have been diagnosed and treated for six weeks as one of malarial remittent fever. Also, a case of pelvic cellulitis, notwithstanding the local pain, was diagnosed as malaria. In such cases, however, a correct, solution of the case is generally arrived at before any serious trouble arises. But an error of this kind in the diagnosis of typhoid fever would, at least in my hands, prove fatal. We should bear in mind that, notwithstanding the teachings of our professors, typhoid fever does exist in this community, and to such an extent that it is time for us to eradicate from our minds old prejudices so thoroughly instilled in past years, for there can no longer be a doubt of its existence, and to the extent that we should be prepared to meet at it any time.

Is it not possible that many of the so-called cases of typho-malaria are modified forms of typhoid? May not climatic influences moderate the disease, for certain it is that few, if any, typical cases have ever originated in our city. Malarial and typhoid fevers are distinct forms and easily diagnosed, making the treatment clear. But another form of continued fever exists, so different in its origin, its symptoms and its cause from either of these two forms that I have deemed it my duty to introduce the subject here to-night and to ask the members of this Society to give the matter their time and consideration. This fever, so I have observed, is usually ushered in without any premonitory symptoms. The patient usually complains of feeling dull; the tongue may or may not be coated; the bowels costive, loss of appetite; sometimes nausea and slight elevation of temperature; seldom any functional disturbance. The temperature will vary from 99 degrees to 103 degrees, and the fever will continue from three to nine weeks. Patients are not greatly exhausted by the fever and are sometimes able to keep about their work. One case which I had under treatment, a robust person, was never compelled to lie down during the day, but spent the time in a large arm-chair. The fever lasted ten weeks. In this case, I believe its long continuance was partly due to malnutrition. Nausea complicated the treatment, and the question of treatment became a serious problem, until I had recourse to raw eggs and sherry; this constituted the nourishment during the day, the patient taking as many as half a dozen eggs in twelve hours. At night I gave mulled milk, which proved a soothing and nutritious beverage. Through the entire course of her illness I was at a loss to detect any one symptom sufficiently marked to point to a diagnosis. If malarial, it was not typical in appearance.

For some time I was inclined to believe it septic, but a most careful examination of every organ in the body, repeated several times, failed to substantiate this belief. As regards treatment, I have employed every form known to science without success. Quinine is inert, and antipyretics give but temporary relief. The case will run its course and must be treated symptomatically. My method of managing these cases has always been purely symptomatic not expectant—relieve pain, regulate the bowels, reduce the temperature, tempt the appetite and trust to luck.

If there are any present whose misfortune it has been to meet with such cases, I trust the results have been more gratifying, and that they have settled upon a diagnosis and a formulated plan of treatment.

I have prepared to-night, to present for your consideration, a number of charts showing the course of the various forms of continued fevers rather than monopolize your time with a detailed account of these cases. I will now submit these charts, which I have borrowed from the New Orleans Sanitarium, believing they will prove of some interest.

[After Dr. Martin had read his paper he presented charts representing the three types of fevers of which he had spoken—malarial, typhoid, and the particular type of remittent fever of which he had just spoken, which he said some of his confrères of the country called "slow fever." After the charts had been examined by the gentlemen present Dr. Martin said that as the subject of typhoid fever had been brought up at the last meeting, he had with him a chart of a typhoid case, regarding which he did not think it amiss to say something. The patient had been brought from Lutcher, a sawmill town some distance above New Orleans. Was taken sick ten days before being brought to the Charity Hospital, where he was received May 21, and the case diagnosed as remittent fever. On May 27 he was admitted to the sanitarium. Up to that time had been taking quinine, and did so after the case was diagnosed as typhoid fever. He became delirious on the 28th and remained so for ten days. Pulse was very rapid and there were marked sordes, a thing you do not often see in remittent fever. Bowels very loose, with bloody stools. The treatment was symptomatic. A little antifebrine was given in whisky to reduce the temperature—given in simple powder it distressed the patient too much. Strychnine was given as a tonic—that was really the treatment all the way through—antipyretics and strychnine, and kept on a milk diet. I believe, too, some ten drops of hydrochloric acid were given three times a day. Hemorrhages were treated with injections of laudanum, which seemed to have a good effect.]

### DISCUSSION.

DR. SEXTON said: Dr. Martin has presented a very interesting subject at a very opportune time. The long continued fevers are on the increase in New Orleans, and every practitioner has more or less of such cases to deal with throughout the entire year. When a student at college I remember that Dr. Bemiss, in discussing this subject, taught us that two separate and distinct poisons or morbid processes might exist in the system at the same time, each one modifying the other in its clinical history, so as to make a hybrid disease which was neither clear-cut malaria nor the classical typhoid of the older authors. During my practice in Mississippi, as well as in New Orleans, I have had considerable to do with these long continued fevers or the so-called typho-malaria of Wood. I have always considered it a mixed poison of the malarial element, whatever that may be, plus some septic infection.

The majority of cases coming under my observation presented a mixed clinical history, the malarial element of every-other-day exacerbations, while the septic and enteric symptoms were overshadowed or masked by the more powerful malarial poison. In other cases just the reverse was true.

In my experience with disease I have noticed that real, clear-cut diagnosis or typical symptoms of typhoid are the exception and not the rule. In a considerable experience with typhoid fever, I hardly recall a single case in which all the symptoms of the older writers on typhoid fever are to be found. Either the tongue does not present the characteristic symptoms, or diarrhea or petechial spots are absent.

May it not also be true that malaria may become modified by the hygienic surroundings and changed conditions of the development of our city? If we were all practical microscopists when such cases come under our care, before beginning the treatment we should examine the blood for Laveran's plasmodium malarial bodies, which, being found (if they are the real cause of malaria), would clearly indicate an anti-malarial course of treatment, which would be quite different if we should find the Eberth's bacillus. But whether these microscopic changes or bacilli are the causes or the results of the disease is not clear, at least to some of us.

I have often been puzzled in trying to think out the etiological process of this disease. Investigators tell us that one case of typhoid fever must originate from another, that the dejecta and infected water play the principal part in the spread of typhoid fever, so just how to account for the typhoid condition in these long-continued fevers, having no connection whatever with other cases of fever, has been very puzzling to me. Not more so, however, than the resistance of these fevers to the action of quinine, though they possessed a distinctive intermittent clinical history—better one day and worse the next. That the fever has a malarial element in it is proven by the fact that it is most prevalent in malarial districts with every-other-day exacerbations. I have always considered it a non-contagious disease, having seen numerous cases where only one member of a large family had been affected with it. In its morbid anatomy it partakes both of the nature of typhoid and malarial fever. In substantiation of the fact that it is malarial fever, plus some other septic infection, I will call the attention of the Society to the fact that it is most frequently found where such unhygienic conditions as overcrowding, bad ventilation, sewer gas, bad food and fruits from the market are used. In our city practice, perhaps, sewer gas more often represents the plus element in the etiology of the fever than any other of the causes mentioned.

With regard to its treatment I have usually met symptoms as they arose. Constipation in the beginning of the fever is the rule; to combat this I give from two and one-half to five grains each of calomel and soda at bedtime, provided the patient has never been salivated. In this event I use phosphate of soda, cascara sagrada or some vegetable pill. Later on in the disease I am more cautious about the use of purgatives, and have found good results by flushing out the bowels with antiseptic douches. Pyrexia has to be combated, and I have usually accomplished this by sponging with tepid or cold water, rolling in wet pack and giving freely of cold water both by mouth and rectum, if agreeable to the patient. When the fever is high I usually keep the head enveloped in iced towels. An admirable diaphoretic mixture is made up of tincture of aconite, spirits of nitre and syrup of squills. More recent remedies for pyrexia belong to the coal tar group, one of which is about as efficacious as the other. I only give such remedies when the temperature is high, usually combining them with a toddy, and not repeating very often.

For the tonic effect of the quinine I usually give three capsules daily, combined with acetanilide or phenacetine to prevent disagreeable head symptoms and to meet the neuralgic element in the disease. I place more dependence upon the sponging and cold baths, however, than any internal medication.

Large doses of quinine, while they may act as an antipyretic, will not abort the fever, and are damaging to the nervous system, hence should not be tried. As an intestinal antiseptic I use the emulsions of turpentine with salol or salicylate of bismuth; camphorated tincture of opium is added if there is exhausting diarrhea. Internal hemorrhages are best controlled by some preparation of opium, and the ice bag. I have had good results from phosphomuriate of quinine compound with arsenic, as a general tonic, repeated four or five times daily.

Dieting is very important, and should be confined to milk, broths, soups and gruel, nourishing in quality small in quantity, often repeated. Under this plan of treatment I have had perhaps a mortality of about four per cent, in the treatment of a large number of cases.

DR. CHASSAIGNAC: I wish first, when it comes to a question of fact, to record my experience in rather a different way from that of Dr. Sexton. I have very frequently seen here in New Orleans typical cases of intermittent fever, either of the quotidian, the tertian, or even the quartan type. I have seen them very clear cut, and in people who had not been out of the city at all; who had marked chill, and had it come back the next day, or the day after, at the same hour. Again, neither of these cases has any connection with the other form of the fever of which the doctor states it is his experience that it occurs where the hygienic surroundings are poor and the food bad. I have seen these cases in some of the best families in the city—people who live well, who are cleanly, and whose houses are good and well ventilated, and not troubled any by sewage.

Now when it comes to the discussion of this long-continued fever, I think we want to decide first what we want to call typhoid fever. Many of us have seen cases that had been recognized as only simple continued fevers, so-called, as far as the symptoms were concerned, including the range of the temperature, and having nothing at all typical of typhoid fever, when the patient died, as in some cases I have in mind at the Charity Hospital, and a post mortem examination was made, reveal what we are taught to consider the characteristic lesion of typhoid fever, as far as Peyer's patches are concerned. So we have to decide whether this condition of Peyer's patches necessarily means that typhoid fever has been at work in the system. The two things that we have to determine, then, are (1) what we want to call typhoid fever; and (2) whether we can call typhoid fever only what is put down in the books.

This is why at our preceding discussion I used the term "classical typhoid" fever. Certainly, we have very little of that here, and it is certain also that these fevers we have under discussion are not that, whatever else you choose to call them. If they ever turn out to be due to the same poison as a regular case of typhoid fever, and that poison modified in some way or other, I cannot say. They certainly are not the classical form of typhoid fever. We must determine, then, whether we are to consider as typhoid fever every case that shows the characteristic affection of Peyer's patches, as revealed in post mortem examination; or whether we can call typhoid fever only those cases which in life present what we are taught to consider the characteristic symptoms. If we are to be guided entirely by the nervous symptoms, as it is stated they do in some parts of the world, we would have to exclude the vast majority of these cases at once from consideration. That is one thing very striking, that no matter how high the temperature goes, no matter how delirious the patients may get, they do not experience the typical symptoms of typhoid fever. This is one of the differences experienced; and we also do not have coma vigils, the murmurings and gurglings, nor the typical bloody stools that we have in typhoid fever; so if it were not for that one point, where the lesion of Peyer's patches has been detected at post mortem, I would state unhesitatingly that we could not consider such a case to be typhoid fever, because the run of the temperature is different, the character of the stools is different, and the symptoms, so far as the nervous system is concerned, are different; therefore, if it were not for that one point we could exclude it altogether.

When it comes to treatment of this fever, whatever you choose to call it—long-continued, remittent, or anything you please—I believe it is treated very easily, and with very little medication if attention is given to the feeding of the patient. By feeding I do not mean crowding food upon him, but eliminating the indigestible from his diet and allowing him only the liquid or easily assimilated articles. Reduce the temperature by means of baths as well as antipyretics. Under such treatment I believe a large majority of such cases will get well.

---

### A MODIFIED OPERATION FOR THE RELIEF OF UNDESCENDED TESTIS.

Bidwell, in the *Lancet*, June 17, 1893, describes his method of operating for undescended testicle. An incision is made the same as for the radical cure of hernia, except that it is not carried into the scrotum. When possible, the testicle is squeezed out of the canal, but when this cannot be done, the fibers of the external oblique have to be divided. The tunica vaginalis is then opened and divided just above the testis; if a hernia be present its upper end is dissected up, and after being ligatured at the internal ring cut off; when no hernia is present it is simply cut away. The fibres of the cremaster are cut across and the testicle is pulled away from the cord whilst the structures between the vas and the epididymis are carefully divided with the point of the knife. In this way the testicle becomes inverted, the vas entering at the top instead of at the bottom of the epididymis. During the separation the spermatic artery, veins and nerves are divided and require ligature, but the artery of the vas, of course, is carefully saved. The finger is then pushed from the incision down into the scrotum, and a needle, with an eye at its point, carrying a silk ligature, thrust upward. The needle is unthreaded and passed through the extremity of the testis, then again threaded and withdrawn, thus leaving the ligature attached by its loop to the testis, and its two free ends hanging externally. These ends are fastened, by means of a rubber tube, to a wire cage, and thus constant traction is made, which drags the testis well down into the scrotum, usually by the third day. The inguinal canal is closed by Macewen's method. Three cases are given in detail, and mention is made of Watson-Cheyene having used a similar procedure.—*University Medical Magazine.*

